# Large language model approach to uncover reasoning patterns in forensic psychiatric assessment

**DOI:** 10.1038/s41598-026-53275-z

**Published:** 2026-05-23

**Authors:** Johannes Lieslehto, Jari Tiihonen, Markku Lähteenvuo, Allan Seppänen

**Affiliations:** 1 Department of Forensic Psychiatry, University of Eastern Finland, Niuvanniemi Hospital, Kuopio, Finland; 2 Department of Clinical Neuroscience, Karolinska Institutet, Stockholm, Sweden; 3 Institute for Molecular Medicine Finland, University of Helsinki, Helsinki, Finland; 4 Center for Psychiatry Research, Stockholm City Council, Stockholm, Sweden

**Keywords:** Forensic psychiatry, Criminal responsibility, Schizophrenia, Large language models, Explainable AI, Decision-making, Criminology, Psychology, Psychology

## Abstract

Forensic psychiatric assessment (FPA) evaluates whether mental disorders impair an individual’s criminal responsibility, yet the reasoning underlying these high-stakes judgments remains largely implicit and susceptible to subjectivity. We investigated whether large language models (LLMs) can quantify and elucidate reasoning patterns embedded in FPAs, and which components contribute most. We analyzed 253 FPAs conducted at a Finnish forensic hospital between 2018 and 2023. Text from each report section was embedded using a Finnish Sentence-BERT model, and section-specific embeddings were classified with support vector machines within a nested cross-validation framework. Model performance was evaluated using the area under the receiver operating characteristic curve (AUROC), complemented by word- and sentence-level explainability analyses. Among individual sections, the psychiatric evaluation (AUROC 0.90, 95% CI 0.86–0.94), psychological assessment (0.88, 0.84–0.92), and previous records (0.83, 0.78–0.88) were most predictive of criminal responsibility. Combining multiple sections further improved discrimination (0.94, 0.90–0.97). Linguistic markers related to psychosis, disorganization, and psychiatric treatment were associated with criminal irresponsibility, whereas substance use, antisocial traits, and organized behavior were more frequent among those deemed responsible. These findings demonstrate that LLM-based methods can identify and quantify textual patterns associated with forensic psychiatric decision-making.

**Supplementary Information:**

The online version contains supplementary material available at 10.1038/s41598-026-53275-z.

## Introduction

Forensic psychiatry stands at the intersection of psychiatry and the law, tasked with answering complex questions of criminal responsibility, capacity, and risk^1–3^. A central principle recognized across most legal systems is that individuals with severe mental disorders, particularly schizophrenia and other psychotic illnesses, may be deemed criminally irresponsible when their symptoms causally affect behavior or understanding at the time of the offense^3,4^. Forensic psychiatric assessment (FPA) integrates diverse sources of information, such as structured psychiatric evaluations, cognitive testing, neurological examination, collateral records, behavioral observations and legal documentation, into a single narrative that informs judicial decision-making^3^.

Although expert judgment in FPA remains the gold standard, its reliance on multiple complex data sources can render the reasoning process opaque, idiosyncratic, and difficult to reproduce. In practice, it is challenging to delineate in detail how a given conclusion was reached, which factors most strongly influenced the conclusions, and whether alternative interpretations might have emerged had certain findings been further corroborated. This challenge is particularly relevant given the high stakes of FPA and the well-documented cognitive biases inherent in human judgment^5–7^. Alarmingly, in a survey spanning 39 countries, 79% of forensic mental health professionals reported concerns about the influence of cognitive bias on such evaluations^8^. These challenges can be further understood within dual-process theories of cognition, which distinguish between fast, intuitive forms of reasoning and slower, more analytical evaluation^9^. In the context of forensic psychiatric assessment, these processes may jointly shape clinical judgments, contributing both to efficient integration of complex information and to potential sources of biases such as anchoring or confirmation bias^5–7^.

The emergence of large language models (LLMs) offers new opportunities to address these challenges^10^. Several transformer-based variants have recently been applied in psychiatric diagnostic and prognostic tasks with promising results^11–14^. Beyond these applications, LLMs also provide a unique avenue to examine the latent reasoning embedded in narrative clinical documentation. By identifying the linguistic features that drive human reasoning, LLMs can “reverse engineer” clinical intuition and make explicit the implicit heuristics that guide expert judgment. For example, a recent study on autism diagnostics demonstrated that while the DSM-5 criteria emphasize social communication deficits, clinicians’ narrative reports focus disproportionately on repetitive behaviors as central to their diagnostic impressions^15^. In forensic psychiatry, LLMs have been used for information extraction and summarization^16^ but, to our knowledge, not to elucidate human reasoning patterns in evaluation and decision making.

In this study, we applied LLMs to FPA reports conducted at Finland’s largest forensic psychiatric hospital, with the aim of elucidating the reasoning processes underlying determinations of criminal responsibility. Specifically, we sought to identify which components of the evaluation most strongly influenced clinical decision-making and to compare their relative predictive value. In addition, we implemented interpretability analyses to highlight the textual features and sections in the report most critical to the examiners’ conclusions. Given the absence of prior empirical work on the relative contribution of different components of forensic psychiatric assessments to determinations of criminal responsibility, our approach was primarily exploratory. However, based on established clinical and legal principles^3,4^, we expected that linguistic features reflecting psychotic symptoms and their functional impact would be associated with findings of criminal irresponsibility. The overarching aim of the present study was to increase transparency in forensic psychiatric reasoning by making patterns in documented assessments more explicit and quantifiable, which may, in the future, help address biases in decision-making in FFA.

## Methods

### Data collection and cohort

The workflow describing the analyses of the present study is presented in Supplementary Fig. 1. The authors assert that all procedures contributing to this study comply with the ethical standards of the relevant national and institutional committees on human experimentation and with the Helsinki Declaration of 1975, as revised in 2013. The study protocol was approved by the institutional review board of Niuvanniemi Hospital. Since the study relied on registry data and involved no direct contact with participants, informed consent was not required under the legislation of Finland. We followed TRIPOD-LLM reporting guidelines throughout the study^17^.

In Finland, forensic psychiatric examinations are highly structured and regulated within a centralized national evaluation system^18^. Annually, approximately 80–100 examinations are performed in the entire country, typically on an inpatient basis, at the state forensic hospitals Niuvanniemi or Vanha Vaasa, or in specialized regional forensic units. The process, which usually lasts ca. two months, involves a multidisciplinary team including psychiatrists, psychologists, nurses, and social workers and culminates in a report that addresses criminal responsibility, the need for involuntary treatment, and the defendant’s capacity to exercise their right to be heard at trial.

A detailed description of the Finnish FPA process has been described in detail elsewhere^18^. Briefly, under Finnish law, courts may order a forensic psychiatric examination if the accused is alleged to have committed a punishable offense, the evaluation is justified, and the accused consents, is detained, or faces imprisonment exceeding 1 year. Examinations may also be authorized by courts of appeal or during pretrial investigations. The primary legal question is criminal responsibility: individuals are not responsible if, at the time of the offense, they were unable to understand its nature or unlawfulness or to control their behavior because of mental illness, severe cognitive deficiency, or other comparable mental disturbances. Finnish law also recognizes diminished responsibility, which applies when the individual’s capacity for understanding or behavioral control is substantially impaired but not abolished^18^.

We retrospectively identified 253 FPA reports completed at Niuvanniemi Hospital, the largest forensic psychiatric hospital in the Nordic countries, between January 2018 and December 2023. All FPA reports were deidentified prior to analysis, data access was restricted to authorized study personnel only, and all analyses were done on a local computer administered by the hospital.

The primary outcome label was “criminally responsible” versus “criminally irresponsible,” as documented in the official report. The secondary labels included “diminished responsibility” and psychiatric diagnoses (e.g., schizophrenia spectrum disorder, bipolar disorder), which were extracted for exploratory analyses.

### Text processing pipeline

Analyses were performed via Python version 3.11 and R version 4.4.2. Each FPA report was divided into 9 semantically coherent sections, which were modeled separately: (1) criminal records; (2) legal documents (including descriptions of the criminal act, toxicology reports, pretrial materials, and court documents); (3) previous records (e.g. questionnaires for relatives, prior hospital records, military service documents, social service files, and prison health care notes); (4) personal history (self-described history of own life); (5) physical examinations during the FPA (e.g., physician’s examination, laboratory tests, electrocardiography, and brain imaging); (6) psychological assessment (e.g., cognitive testing and personality inventories); (7) psychiatric assessment (structured diagnostic interviews such as the SCID and narrative formulations); (8) inpatient nursing observations; and (9) conclusions (integration of all components into a judgment regarding mental state at the time of the offense). A more detailed description of each section is provided in the Supplementary Table 1. Parsing was conducted automatically via regular-expression heuristics, followed by manual review of the section headers to ensure accuracy.

### Text embedding

Each of the 9 text sections was embedded via the Finnish version of Sentence-BERT (SBERT; TurkuNLP/sbert-cased-finnish-paraphrase), a transformer-based pretrained encoder model that produces sentence-level embeddings^19–21^. To preserve semantic coherence in long passages, the sections were divided into overlapping chunks (maximum length 512 tokens; overlap of 1 sentence). Each chunk was processed through SBERT, and mean-pooled embeddings from the final layer were extracted. Chunk embeddings were then combined via token-length–weighted averaging, yielding a single 768-dimensional vector per section per report for downstream classification. The resulting 768-dimensional representation can be considered a high-dimensional latent space that captures the semantic content of the text^22^. As a robustness check, we also evaluated 2 alternative pretrained transformer models: Finnish BERT (a base BERT model pretrained exclusively on Finnish corpora)^19–21^ and XLM-RoBERTa (a multilingual transformer pretrained on more than 100 languages)^23^.

### Machine learning classification

Text embeddings from each text section were used as features in a support vector machine (SVM) classifier with a radial basis function kernel^24^. For this purpose, we applied functions from the mlr and caret packages^25–27^. SVM does not rely on classical distributional assumptions but instead depend on the structure of the feature space and the choice of kernel function, which defines the form of the decision boundary^28^. Separate models were trained and tested for each section (e.g., psychiatric assessment, personal history). To prevent information leakage^29^, all analyses were performed within a nested cross-validation framework (10 outer folds with 20 repetitions and 3 inner folds for hyperparameter tuning), which incorporated scaling and centering as preprocessing steps. The hyperparameters (*C* [2⁻⁴, 2⁻²,…, 2⁶, 2⁸] and γ [2⁻¹⁴, 2⁻¹², …, 2⁻², 2⁰]) were optimized via grid search. An identical cross-validation structure was used for training and testing each of the nine section embeddings. Performance was measured via the area under the receiver operating characteristic curve (AUROC) of the SVM output probabilities (50% used as a threshold for the outcome classes). Briefly, an AUROC of 1.0 indicates perfect discrimination and 0.5 corresponds to chance-level performance. The results for each section embedding are reported as the mean out-of-training predictions across outer folds that were used for drawing ROC curves. P values for the discrimination performance of each section embedding were derived from a permutation analysis of 1000 iterations, and Benjamini–Hochberg false discovery rate (FDR) correction was applied to reduce false positives^30^. To ensure robustness and model-agnostic validity, identical analyses were repeated via embeddings derived from Finnish BERT^19–21^ and XLM-RoBERTa^23^, as well as via elastic net regression (details in the Supplement).

Finally, to explore the optimal combination of information across different text sections of the FPA and to identify components that did not contribute additional predictive value to determinations of criminal responsibility, we implemented an ensemble learner that integrated the outputs of all nonconclusion sections. Sequential backward selection (SBS) was applied^31^, while all other aspects of the previously mentioned nested cross-validation structure were maintained. The performance of this ensemble learner was compared with that of the conclusions model and the best-performing individual sections via De Long’s test for correlated ROC curves^32^, and the selection frequencies of individual sections were visualized.

### Explainability analyses

To examine which textual elements most strongly influenced SVM classifier decisions, we implemented 2 complementary interpretability strategies designed to reveal lexical patterns underlying model predictions beyond “black-box” embeddings.

First, we applied a lemmatization pipeline to normalize the inflected Finnish word forms to dictionary-based forms via the Stanza toolkit that accompanies pretrained Finnish models^33^. Lemmatization incorporates morphological analysis and part-of-speech (POS) tagging, a critical step in Finnish given its rich inflection and compounding. Texts were processed sequentially through tokenization, POS tagging, and lemmatization; only content words (nouns, verbs, adjectives, adverbs, proper nouns) were retained, while function words were excluded. The resulting lemma–by–document matrix was filtered to remove rare lemmas (< 20 individuals). Surviving lemmas were correlated with model-predicted probabilities of criminal irresponsibility via Spearman’s ρ, with false discovery rate correction applied to P values.

Second, we performed a sentence-removal ablation analysis on section-specific models that demonstrated significant predictive value after false discovery rate correction. For each such model, we selected one individual classified as criminally irresponsible with the highest predicted risk and 1 as criminally responsible with the lowest predicted risk. Within the relevant text section for each individual, sentences were iteratively removed, embeddings were recomputed, and the classifier was reapplied. This procedure allowed us to identify sentences whose removal produced the greatest change in predicted class probability.

### Performance stability analysis

We assessed the stability of model discrimination with respect to sample size using a subsampling-based analysis. For each section-specific model, we generated stratified random subsamples of increasing size, starting from *n* = 40 and increasing in increments of 10 up to the full sample size. At each sample size, subsampling was repeated 1000 times to account for sampling variability. For each subsample, AUROC was calculated and the resulting AUROC distributions at each sample size were summarized using the median and interquartile range.

## Results

### Sociodemographic and clinical characteristics

We identified 253 individuals who underwent FPA: 107 were deemed criminally irresponsible, 26 were considered to have reduced responsibility, and 120 were deemed criminally responsible (Table 1). The mean (SD) ages were 35.08 (12.34), 28.15 (12.70), and 34.83 (11.56) years, respectively, and most participants were men (> 80% across groups). Marked diagnostic differences emerged: schizophrenia spectrum disorders predominated in the irresponsible group but were almost nonexistent in the responsible group. The index diagnosis of a personality disorder was substantially more frequent among responsible individuals than among those deemed irresponsible or reduced. Antisocial personality disorder as a primary or secondary diagnosis was observed in 44.2% of the responsible individuals, compared with 15.0% of the irresponsible individuals and 23.1% of the reduced individuals with reduced responsibility. The diagnosis of borderline personality disorder was most prevalent in the responsible group (35.0%) and rare in the other groups. Substance use disorders (as a primary diagnosis or as a comorbidity) were common across all groups (70–81%), without significant group differences.

### Classification performance

**Fig. 1:**
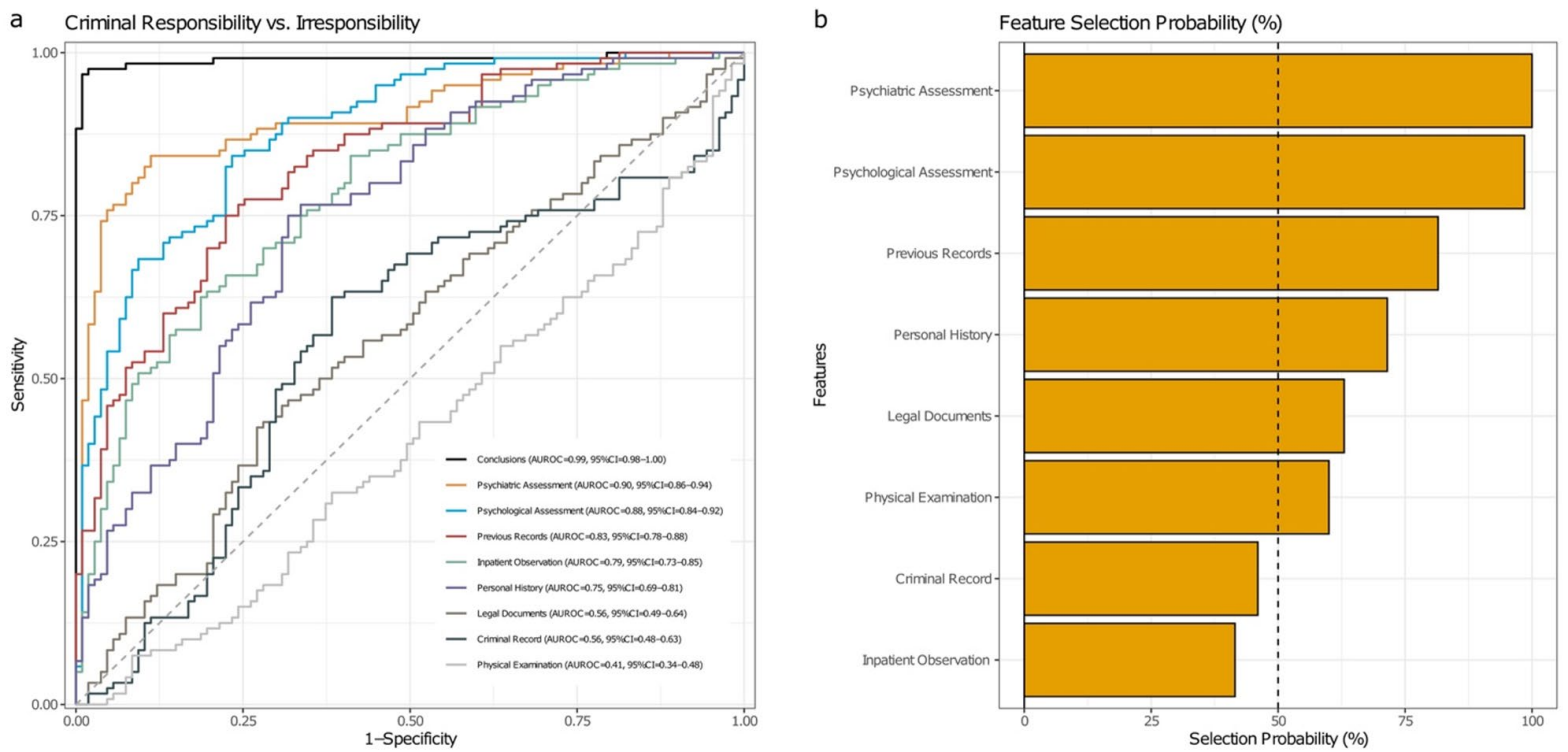
(**a**) Receiver operating characteristic (ROC) curves for Section-Specific Classifiers Distinguishing Criminal Responsibility vs. Criminal Irresponsibility via SBERT embeddings as features in support vector machines. (**b**) The selection probability of each feature in the sequential backward selection wrapper.

In section-specific classification analyses between criminally responsible and irresponsible individuals, performance varied markedly across components of the FPA (Fig. 1a). The highest predictive accuracy for criminal responsibility vs. irresponsibility was achieved via the conclusions section, which provided almost perfect separation between criminally responsible and irresponsible individuals (AUROC = 0.99; 95% CI, 0.98–1.00). Among the individual assessment domains outside of the conclusions, the psychiatric assessment (AUROC = 0.90; 95% CI, 0.86–0.94), psychological assessment (AUROC = 0.88; 95% CI, 0.84–0.92), and previous records (AUROC = 0.83; 95% CI, 0.78–0.88) provided the strongest predictive value. Moderate discrimination was observed for inpatient observations (AUROC = 0.79; 95% CI, 0.73–0.85) and personal history (AUROC = 0.75; 95% CI, 0.69–0.81). In contrast, legal documents (AUROC = 0.56; 95% CI, 0.49–0.64), criminal records (AUROC = 0.56; 95% CI, 0.48–0.63), and physical examinations (AUROC = 0.41; 95% CI, 0.34–0.48) performed below chance level (FDR-corrected *P* > .05). These results were essentially the same even when elastic net regression or other pretrained LLMs were used (Supplementary Figs. 2–4).

SBS analysis revealed that psychiatric and psychological assessments were the most frequently retained sections in the ensemble model, followed by previous records and personal history (Fig. 1b). In contrast, inpatient observations and criminal records were excluded with over 50% probability, indicating a limited additional predictive contribution of these two sections beyond the other six components. Compared with the psychiatric assessment alone, the ensemble model achieved a significantly greater discriminative performance (AUC = 0.94 vs. 0.90; DeLong Z = 2.54; *P* = .01; 95% CI for AUC difference, − 0.07 to − 0.01). Nevertheless, the conclusions model significantly outperformed this ensemble model on the basis of sections outside of the conclusions (AUC = 0.99 vs. 0.94; DeLong Z = 3.48; *P* = .0005; 95% CI for AUC difference, 0.02–0.08).

**Fig. 2:**
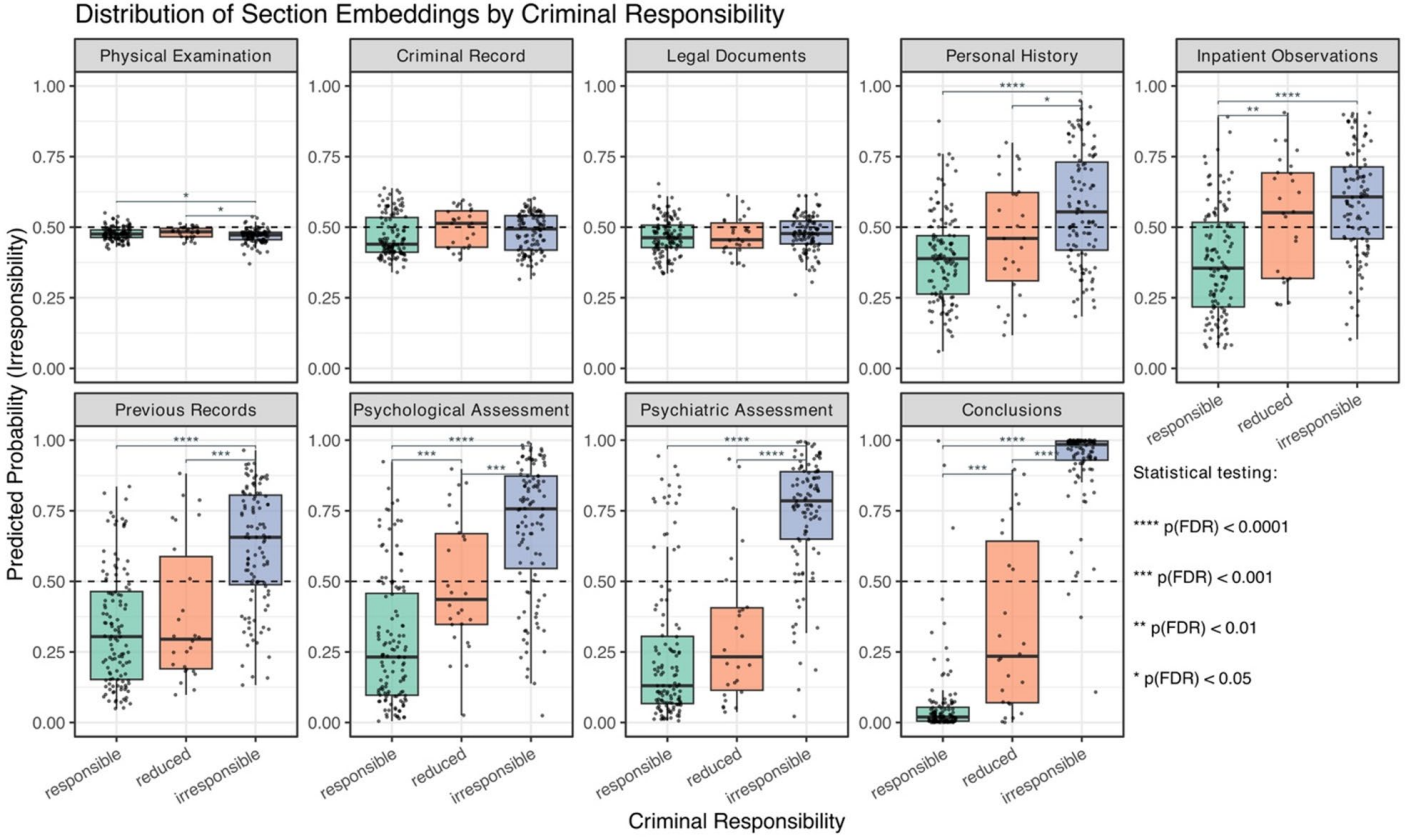
Predicted Probabilities of Criminal Irresponsibility in Different Criminal Responsibility Groups across Forensic Psychiatric Assessment Sections. Boxplots displaying the distributions of the predicted probabilities of criminal irresponsibility derived from section-specific SVM classifiers across diagnostic categories. The dashed line represents a 50% probability (i.e., cutoff between criminal responsibility and irresponsibility). Each panel represents one of the nine sections of the forensic psychiatric assessment (FPA). Higher predicted probabilities indicate greater model-assigned likelihoods of criminal irresponsibility.

When individuals deemed to have reduced criminal responsibility (*N* = 23) were projected into the models trained to distinguish between responsible and irresponsible defendants, their predicted probabilities almost consistently fell between the 2 extreme categories (Fig. 2). Across sections with high discrimination performance, particularly psychiatric and psychological assessments, previous records, and conclusions, the reduced responsibility group showed intermediate probability distributions, which were significantly different from those of both the responsible and irresponsible groups after FDR correction (*P*<.05 for all comparisons). In contrast, criminal records, legal documents, and physical examinations did not clearly separate the three groups.

**Fig. 3:**
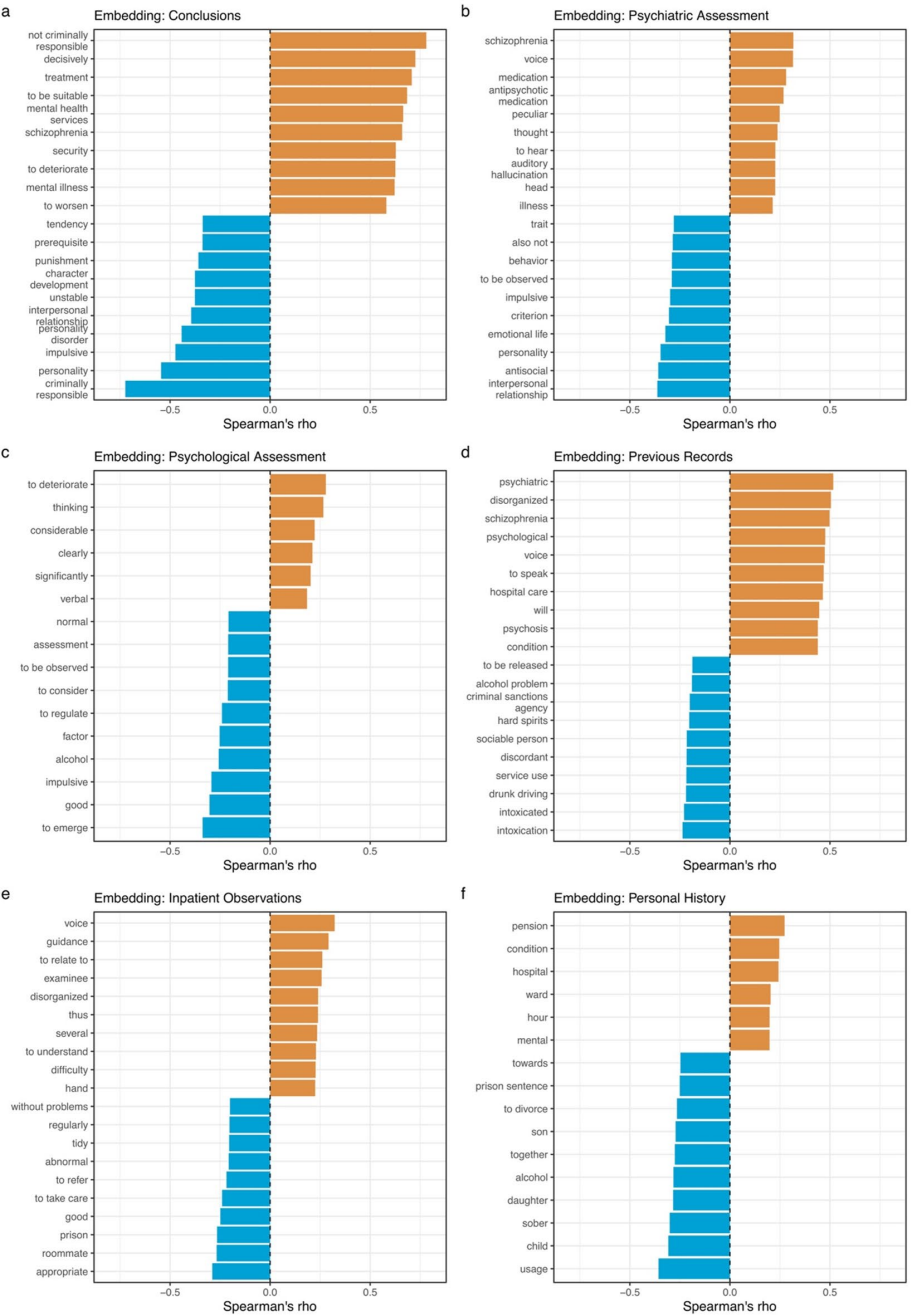
Lemma-Level Correlations with Predicted Criminal Irresponsibility Across Forensic Psychiatric Assessment Sections. The maximum of the top 10 correlations for each direction (i.e., 10 for positive and 10 for negative) within each embedded text section is shown for visualization purposes.

Individuals with schizophrenia spectrum disorders (ICD-10: F20 & F25), the majority of whom were deemed criminally irresponsible, demonstrated the highest predicted probabilities of irresponsibility across nearly all sections, particularly psychiatric and psychological assessments and conclusions (FDR–corrected *P*<.0001, Supplementary Fig. 5). In contrast, individuals with an index diagnosis of personality disorders, or substance use disorders, most often judged as criminally responsible, had significantly lower predicted probabilities of irresponsibility (FDR-corrected *P*<.001). Those with primary diagnoses of affective disorders, developmental disorders, and intellectual disabilities presented heterogeneous profiles, with intermediate predicted probabilities and less consistent separation from the other groups see fig 3.

### Explanation analyses

To explore the linguistic features most associated with determinations of criminal responsibility, we conducted lemma-level correlation analyses within each report section (Fig. 3). In conclusion section, words and phrases such as “schizophrenia”, “mental illness”, and “treatment” were strongly associated with the likelihood of criminal irresponsibility, whereas terms related to personality, impulsivity, and punishment were correlated with full responsibility. In the psychiatric assessment, lemmas describing psychotic phenomena (“auditory hallucination”, “to hear”, “voice”) and treatment were linked to irresponsibility, whereas descriptors of certain personality traits and behavior (“impulsive”, “antisocial”, “interpersonal relationships”) were correlated with responsibility. The psychological assessment similarly revealed associations of words such as “deterioration”, “considerable”, and “thinking” with irresponsibility, whereas terms reflecting normality (e.g., “normal”, “good”), alcohol use, or impulsivity were more frequent among those who were more likely to be criminally responsible. In previous records, indications of alcohol use tended to be associated with responsibility, whereas terms related to psychosis (e.g., “schizophrenia”, “voice”) and psychiatric treatment aligned with irresponsibility. In inpatient observations, descriptors of disorganization (“abnormal”, “disorganized”, “difficulty to understand”) correlated with the prediction of irresponsibility, whereas positive functioning (“tidy”, “good”, “to take care”) was associated with the prediction of responsibility. Finally, in personal history, references to alcohol use and criminal sanctions correlated with responsibility, whereas terms linked to hospitalization or psychiatric care were more frequent among those deemed to be irresponsible. The original lemmas in Finnish are depicted in Supplementary Fig. 6.

To complement these findings, sentence-removal ablation analyses (Supplementary Table 2) identified specific passages within sections that most strongly determined the ML classifier predictions. Passages describing psychotic delusions, disorganized behavior, or psychiatric treatment were highly influential in predicting criminal irresponsibility, whereas descriptions highlighting antisocial conduct, substance use, or maladaptive personality traits tended to drive assessments toward responsibility.

### Performance stability analysis

As shown in Supplementary Fig. 7, median AUROC values remained relatively stable after about the sample size of about 100 individuals for all sections, indicating that model discrimination was not strongly dependent on sample size within the observed range. In contrast, variability in AUROC estimates decreased substantially as sample size increased.

## Discussion

To our knowledge, this study represents the first quantitative attempt to elucidate the structure of forensic psychiatric reasoning, a domain that has historically resisted formal measurement. By analyzing 253 forensic psychiatric assessments, we thoroughly trained and tested^29^ an LLM-based classifier to identify the specific textual components most influential in the determination of criminal responsibility. Our analyses revealed consistent linguistic patterns reflecting, e.g., psychotic symptoms, personality traits, criminal behavior, and functioning, and how these features differentially shift the decision boundary toward criminal responsibility or irresponsibility depending on their degree. The results were highly similar regardless of the encoder model or ML classifier used. AUROC values of 0.88–0.90 for psychiatric and psychological assessments indicate that these sections strongly differentiate between responsibility groups, whereas near-chance performance in legal documents suggests limited relevance for this distinction. Importantly, the aim of this work was not to develop a clinically applicable prediction model, but to provide a mechanistic understanding of how different elements of forensic psychiatric assessments relate to final decisions. Although AUROC values (e.g., ~ 0.90) in some sections indicate strong discrimination, they do not imply error-free classification (false positives and false negatives), which would have serious consequences in high-stakes contexts of FFAs.

National guidelines for forensic psychiatric examinations in Finland, which are consistent with international standards^3,4^, emphasize that determinations of criminal responsibility should be based on whether a mental disorder abolished or substantially impaired the individual’s ability to understand the nature or unlawfulness of the act or to control their behavior^18^. These principles provide a normative framework for how criminal responsibility should be assessed, but they do not specify which information source, clinical features or narrative patterns carry the greatest weight in practice. Our work has shown that it is possible, via the means of LLMs, to make this decision process more measurable and transparent. By systematically identifying linguistic and contextual cues and their influence on determining criminal responsibility, this approach could inform the development of decision-support tools. Ultimately, these tools could help mitigate the well-documented cognitive biases that affect forensic psychiatric evaluations^5,8,34^.

Across the different sections of the FPA, psychiatric and psychological assessments, accompanied by information from previous records, carry the most weight in the decision process. The most consistent text pattern related to criminal irresponsibility encompassed active psychotic symptoms, disorganized behavior and references to current or previous psychiatric treatment. These issues are also emphasized in the conclusions section, where the final decision regarding criminal responsibility is presented and rationalized. Conversely, linguistic patterns that captured personality disorder-related descriptions, substance use, organized and goal-directed actions, and criminal lifestyles were more frequently observed in those who were deemed criminally responsible by the LLM-based classifier. Our findings are consistent with previous natural language processing research on electronic health records for identifying individuals at risk of psychosis, which has shown that incorporating model-identified psychosis-related symptoms from the records improves predictive model performance^11^. Lastly, the identified linguistic patterns likely reflect established legal and clinical criteria for criminal responsibility, as psychotic disorders are widely recognized as central to findings of criminal irresponsibility, whereas personality and substance use disorders are typically insufficient^18^.

The conclusions section, which synthesizes all prior information into a final judgment, yields near-perfect discrimination between responsible and irresponsible individuals. Among the other sections, the psychiatric assessment, psychological assessment, and previous records conferred the highest predictive performance. Combining information from different sections of the FPA reports further improved classification accuracy, suggesting complementary value across these domains. This pattern suggests that forensic psychiatrists rely on the integration of multiple sources of information rather than any single data domain when drawing conclusions. Note, however, that the near-perfect performance of the conclusions section likely reflects, at least in part, that this section explicitly contains the final judgment, introducing a form of information leakage. However, its analysis remains informative for identifying the linguistic features that characterize how such decisions are articulated.

Nevertheless, the performance of this multisection model never matched that of the conclusions section, indicating that the LLM-based classifier used was unable to fully replicate the integrative reasoning process of human examiners when sections were analyzed in isolation. The superior performance of the conclusions section may reflect the uniquely human capacity to utilize tacit knowledge, or phronesis, i.e. weigh and reconcile divergent evidence, read between the lines, calibrate symptom severity against legal standards, and apply normative judgment^35^. This interpretation is consistent with dual-process theories of clinical reasoning, in which intuitive, experience-based processes operate alongside more analytical and structured evaluation of evidence^9^. At the same time, such integrative reasoning processes may also be susceptible to well-described cognitive biases, such as confirmation bias or anchoring, whereby early impressions or salient information disproportionately shape the final judgment^5–7^. In this framework, the conclusions section may capture the output of an integrative process that combines both modes of reasoning, whereas models based on isolated text sections possibly reflect the explicit, analytically accessible component. In contrast to purely explicit, text-based reasoning, tacit knowledge is partially embodied, experience-based, and often inexpressible in explicit form, as “we know more than we can tell.”^36,37^.

Individuals deemed to have diminished criminal responsibility consistently occupied an intermediate position between those classified as fully responsible and those deemed irresponsible, suggesting that the models captured not only categorical distinctions but also gradations of responsibility that mirror clinical and legal reasoning. Across diagnostic groups, similar patterns were observed: schizophrenia spectrum disorders were most strongly associated with findings of criminal irresponsibility, whereas personality and substance use disorders predominantly aligned with determinations of responsibility. Individuals with affective, developmental, or intellectual disabilities demonstrated more heterogeneous profiles, often positioned between these two extremes. The intermediate placement of the diminished-responsibility group is particularly noteworthy, as the model was trained solely on binary labels yet successfully reproduced a continuum consistent with the legal framework of Finnish forensic psychiatry, which recognizes diminished responsibility as a distinct category. In this light, our findings stand in contrast to some other jurisdictions where responsibility is viewed as dichotomous^38,39^.

Several limitations merit consideration. First, our data were derived from a single national context with highly structured forensic assessments; thus, generalizability to other jurisdictions may be limited. Second, although the models identified associations between text and responsibility determinations, causality cannot be inferred. Clinicians’ reasoning is influenced by multiple contextual and interpersonal factors not captured in text alone. Third, this study used pretrained transformer models as text encoders without task-specific fine-tuning. This approach was necessitated by the relatively small sample size. It is also possible that variability across reports limited classifier’s learning, given the modest sample size. Furthermore, the findings may be influenced by biases inherent in clinician-authored reports, and the use of fixed text embeddings may not fully capture the complexity, contextual dependencies, and nuanced reasoning processes involved in FPA. Thus, future studies with substantially larger and more heterogeneous samples, ideally drawn from multiple institutions and jurisdictions, are needed to determine generalizability of different FPA models. In particular, cross-national studies comparing forensic psychiatric assessments across different legal frameworks could help clarify the extent to which the identified reasoning patterns are context-dependent. Future studies incorporating more proximal data, such as raw pre-trial documents, may provide a more direct window into the underlying reasoning process and allow earlier identification of potential biases before they are formalized in the final report. Lastly, the structured and centralized nature of the Finnish forensic psychiatric system may introduce systematic biases in how information is documented, as standardized report formats and shared institutional practices may influence the emphasis placed on specific clinical features.

In conclusion, we showed that LLMs can identify and quantify the textual patterns associated with determinations of criminal responsibility in FPA. Our findings provide a data-driven perspective on how such decisions are expressed in FPA reports, which may contribute to improved transparency and consistency. However, the present approach captures documented reasoning rather than the full underlying decision-making process. Future work should explore the generalizability of these findings across jurisdictions and incorporate more proximal data sources, such as interview transcripts or pre-trial materials, to better examine the structure of forensic reasoning and potential sources of bias.

**Table 1:** Sociodemographic characteristics of the study cohort.

Variable	Irresponsible (*n* = 107)	Reduced (*n* = 26)	Responsible (*n* = 120)	*P*
Sex, No. (%)				
Male	97 (90.7)	21 (80.8)	109 (90.8)	0.283
Female	10 (9.3)	5 (19.2)	11 (9.2)	
Age, Mean ± SD, years	35.08 ± 12.34	28.15 ± 12.70	34.83 ± 11.56	0.025
Diagnosis, No. (%) *				
Schizophrenia spectrum disorders (ICD-10: F20 & F25)	84 (78.5)	<5	<5	< 0.001
Antisocial personality disorder	16 (15.0)	6 (23.1)	53 (44.2)	< 0.001
Borderline personality disorder	<5	<5	42 (35.0)	< 0.001
Substance use disorder	75 (70.1)	19 (73.1)	97 (80.8)	0.164
Affective disorder	5 (4.7)	<5 ()	9 (7.5)	0.193
Developmental disorder	<5	<5	<5	0.309
Intellectual disability	6 (5.6)	<5	<5	0.455

* Primary diagnosis or comorbidity.

Percentages are column percentages within each group. P values reflect group comparisons across all three categories (ANOVA, χ² test, or Fisher’s exact test, as appropriate).

## Supplementary Information

Below is the link to the electronic supplementary material.

Supplementary Material 1

## Data Availability

The data are not publicly available. Researchers can apply for access to these data from the National Board of Health and Welfare by contacting Findata (info@findata.fi).

## Code availability

Codes to reproduce the results of the present study are available at https://github.com/johpulkk/LLM_FPA.
