# Supplementary material for: Large language model approach to uncover reasoning patterns in forensic psychiatric assessment
Source: Sci Rep. 2026 May 23;16:23662. doi: 10.1038/s41598-026-53275-z (PMC13424873; doi:10.1038/s41598-026-53275-z)
Supplement: Supplementary file 1 — Supplementary Material 1 [file 41598_2026_53275_MOESM1_ESM.docx]

**Supplementary Material**

**Large Language Model Approach to Uncover Reasoning Patterns in Forensic Psychiatric Assessment**

**Supplementary Methods**

**Supplementary Table 1-2**

**Supplementary Figures 1-7**

**Supplementary Methods.**

- 1. **Alternative Modeling Strategies**

To ensure robustness and language model–agnostic validity, all analyses were repeated using embeddings derived from both TurkuNLP’s Finnish BERT (bert-base-finnish-cased-v1) XLM-RoBERTa. Finnish BERT is a transformer model pretrained exclusively on large-scale Finnish corpora. XLM-RoBERTa is a multilingual model trained on over 100 languages using a significantly larger CommonCrawl-based dataset. Both models were used to generate text embeddings per section (average over chunks with 128 token overlap between chunks), and the resulting representations were passed to the same SVM classifier and cross-validation framework described in the main text (10 outer folds with 20 permutations, 3 inner folds).

As an alternative classifier to support vector machine, we fit penalized logistic regression with an elastic net penalty. The regularization strength (λ = 0, 0.2, 0.5, 0.8, 1, 2, 5, 10) and mixing parameters (α = 0, 0.2, 0.4, 0.6, 0.8, 1) were selected via grid search via balanced accuracy as the optimization criterion within the nested cross-validation framework (10 outer folds with 20 permutations, 3 inner folds).

| **#** | **Section Name** | **Description** |
| --- | --- | --- |
| **1** | **Criminal Record** | Official record of 10 years prior convictions and sanctions imposed by the Finnish criminal justice system before the index offense leading to FPA. |
| **2** | **Legal Documents** | Procedural documentation directly related to the index offense: description of the criminal act(s), police pretrial investigation materials, toxicology reports, and court-issued orders or judgments. |
| **3** | **Previous Records** | Collateral documentation predating the index offense, including prior hospital or outpatient psychiatric notes, social services reports, military or prison health dossiers, and educational or employment records. |
| **4** | **Personal History** | The examinee’s own autobiographical account of developmental background, family relationships, education, employment, military service, and earlier life experiences. |
| **5** | **Physical Examination** | Somatic assessment conducted during the FPA, including neurological examination, laboratory results, ECG findings, and neuroimaging. Identifies comorbid medical or neurological conditions. |
| **6** | **Inpatient Observation** | Continuous behavioral observations by nursing staff during the inpatient evaluation, covering daily functioning, interactional style, impulse control, emotional regulation, and adherence to ward routines. |
| **7** | **Psychiatric Assessment** | Structured and narrative psychiatric evaluation, including mental status examination, SCID or comparable diagnostic interviews, and lifetime psychiatric history. Represents the core clinical formulation. |
| **8** | **Psychological Assessment** | Standardized tests (e.g., intelligence, memory, executive function, personality inventories) providing a profile of personality and possible psychiatric symptoms, and cognitive and executive functioning. |
| **9** | **Conclusions** | Integrative expert judgment synthesizing all prior sections into a legal-psychiatric opinion on mental state at the time of the offense, level of criminal responsibility and possible treatment requirements. |

**Supplementary Table 1. Description of the different sections of the forensic psychiatric assessment**

FPA=Forensic psychiatric assessment

**Supplementary Table 2 Representative Sentences Identified by Sentence-Removal Ablation Analyses as Highly Influential in Predicting Criminal Irresponsibility and Responsibility Across Forensic Psychiatric Assessment Sections**

| **Section** | **High Relevance in Predicting Criminal Irresponsibility** | **High Relevance in Predicting Criminal Responsibility** |
| --- | --- | --- |
| **Personal History** | “According to the examinee, he hardly has any physical illnesses, only schizophrenia, and the medication is helpful for him.” | “In recent years, the examinee has made his living from unemployment and social assistance benefits as well as from selling drugs.” |
| **Psychiatric Assessment** | “He reported perceiving that behind television discussions there was some larger organization that produced false and provocative information for the discussions.” | “There had been cocaine use at parties.” |
| **Psychological Assessment** | “These were particularly manifested as psychotic delusions guiding his actions, speech saturated with loose associations and illogical reasoning, and inadequate emotional reactions.” | “In the personality assessment, rigidity in morality and interpersonal relationships emerged at times as a trait that moderately impaired his adaptation.” |
| **Inpatient Observations** | “During interactions and in receiving guidance, the examinee had difficulties understanding what was said, and things had to be repeated several times.” | “The examinee arrived for the forensic psychiatric assessment from prison, escorted by guards.” |
| **Previous Records** | “The examinee had been started on the antipsychotic olanzapine, and temazepam as a sleeping medication if needed.” | “The examinee had been treated surgically for a fracture in his hand.” |
| **Conclusions** | “Due to his mental illness, the examinee needs involuntary psychiatric hospitalization, as not committing him to treatment would substantially worsen his mental illness and seriously endanger his own health and safety, as well as the health and safety of others.” | “Negative experiences, such as punishments, do not easily change such behavior – for example, the examinee has continued his substance use despite being taken into custody and placed in a substance rehabilitation unit.” |


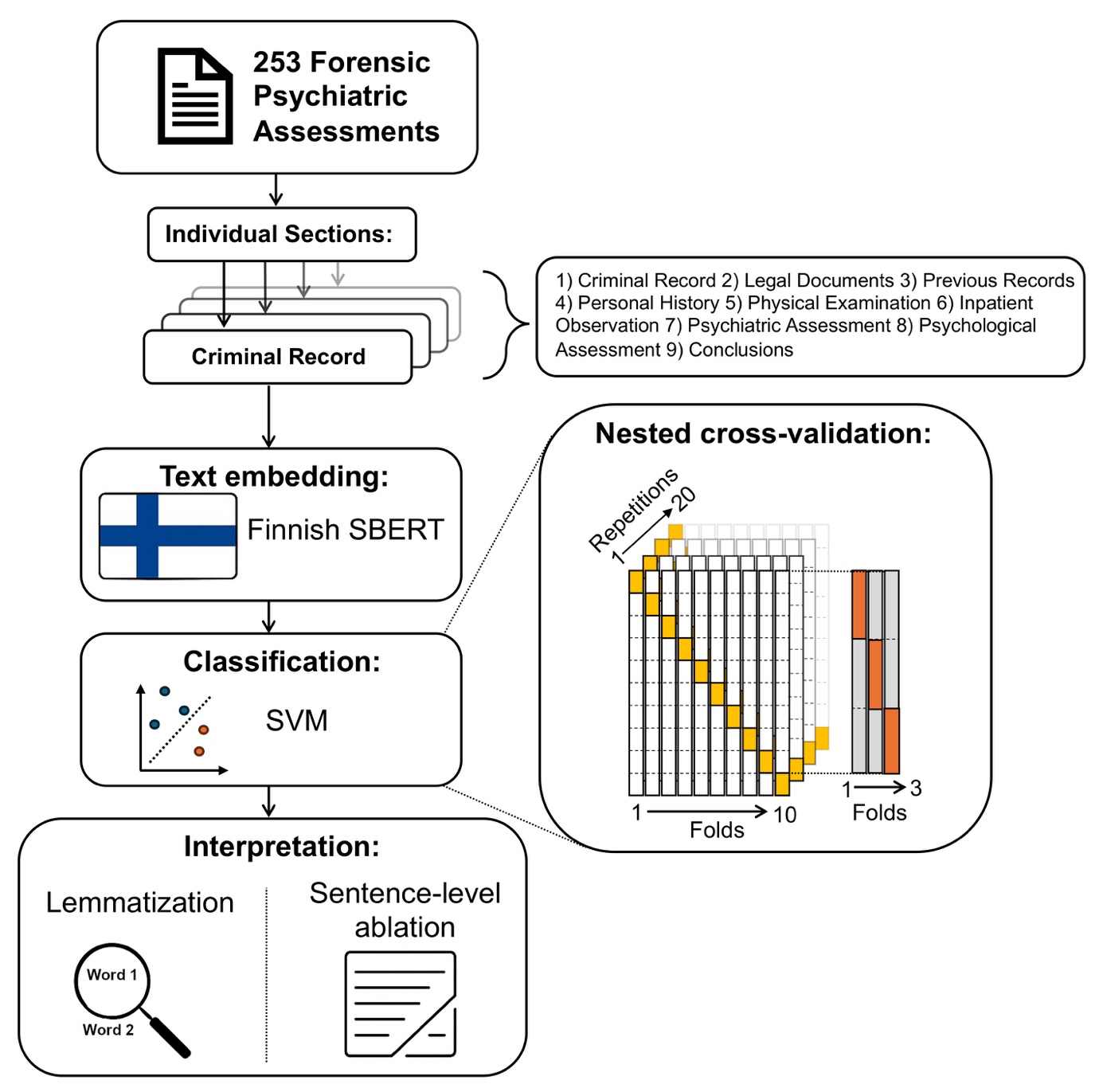


**Supplementary Figure 1.** **Flowchart depicting the analyses of the present study.**

**
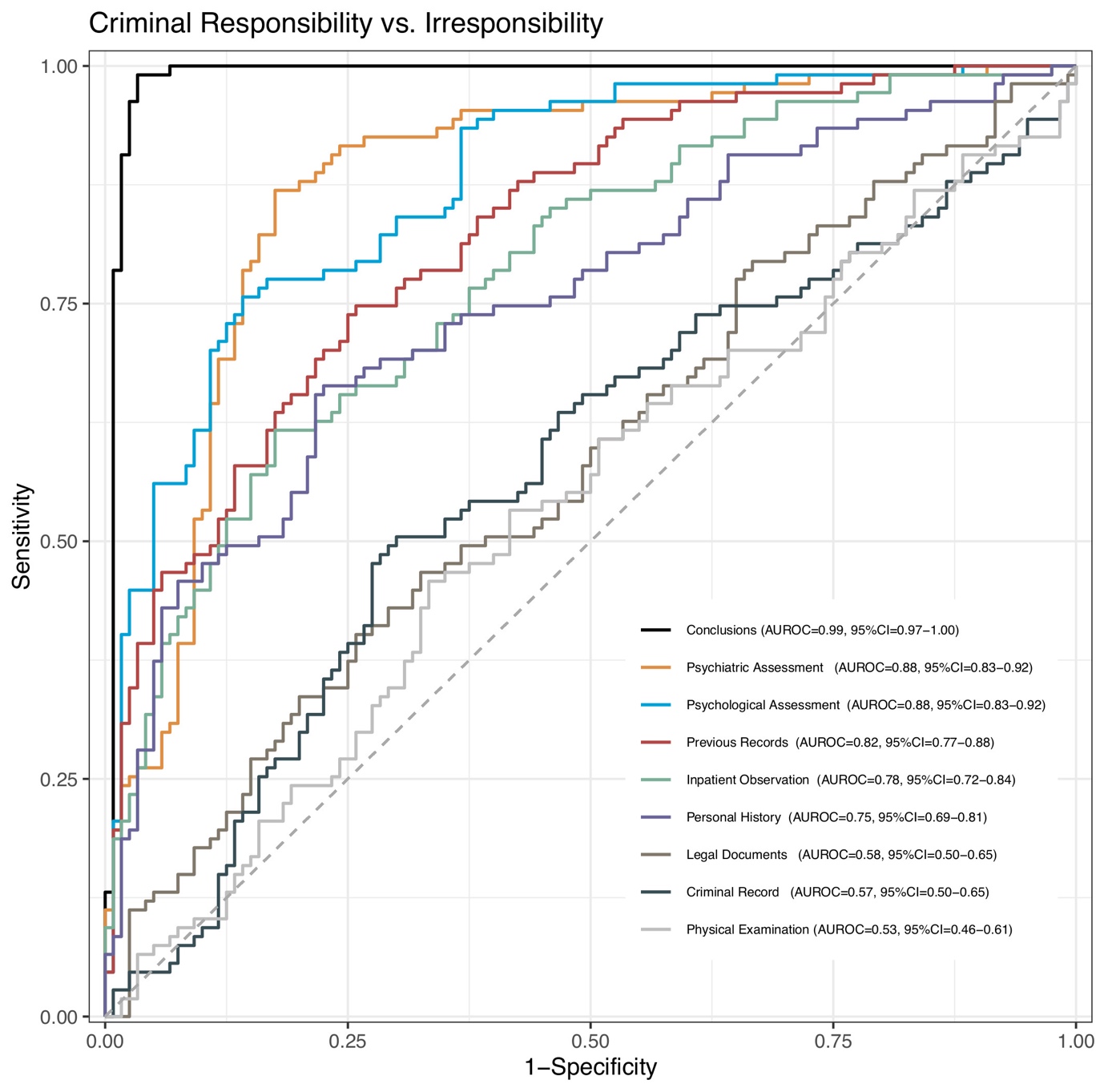
**

**Supplementary Figure 2. Receiver Operating Characteristic (ROC) Curves for Section-Specific Classifiers Distinguishing Criminal Responsibility from Criminal Irresponsibility via SBERT Embedding as Features in Elastic Net Regression.**

**
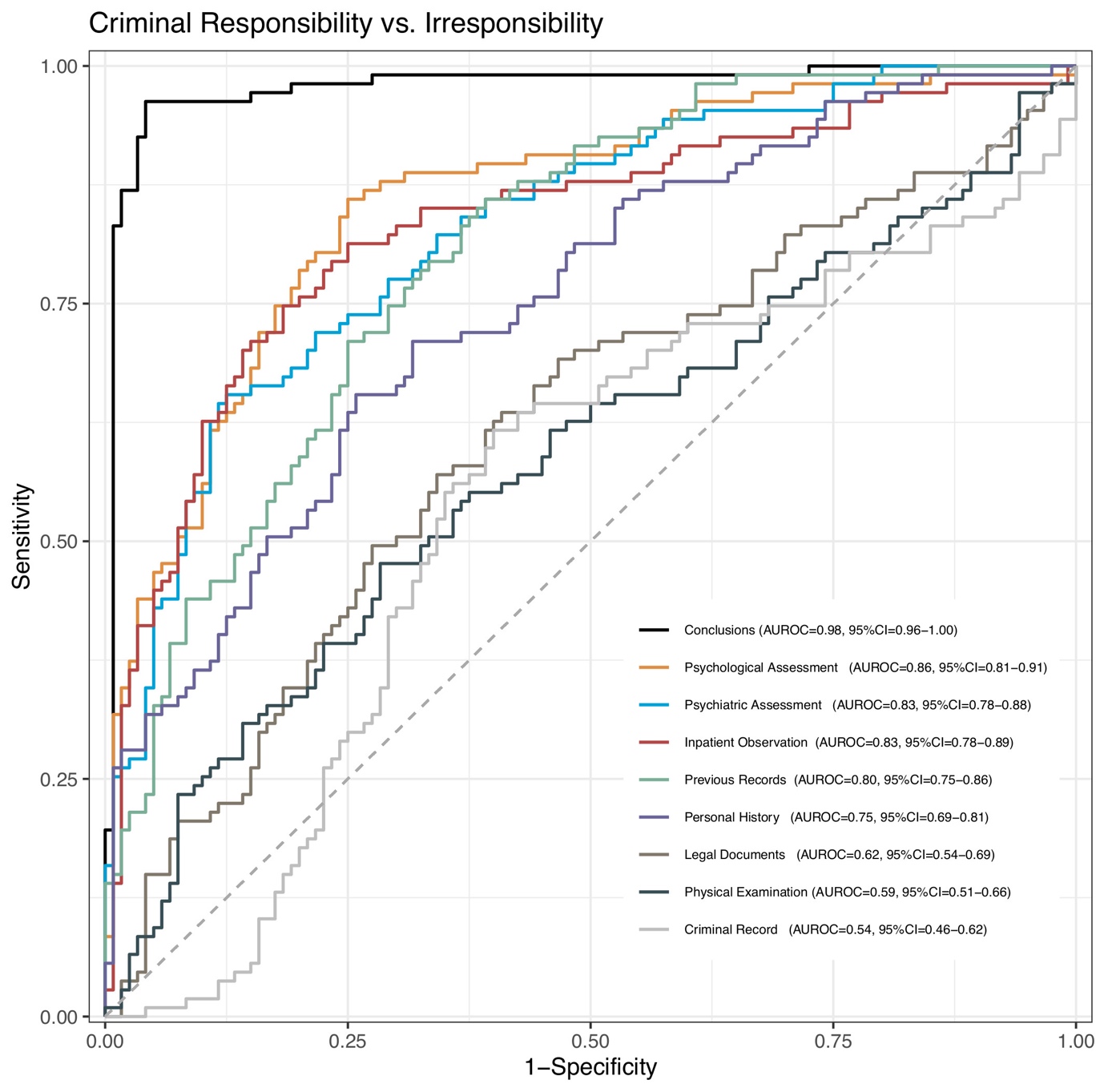
**

**Supplementary Figure 3. Receiver operating characteristic (ROC) curves for section-specific classifiers distinguishing criminal responsibility from criminal irresponsibility via Finnish BERT embeddings as features in SVM.**

**
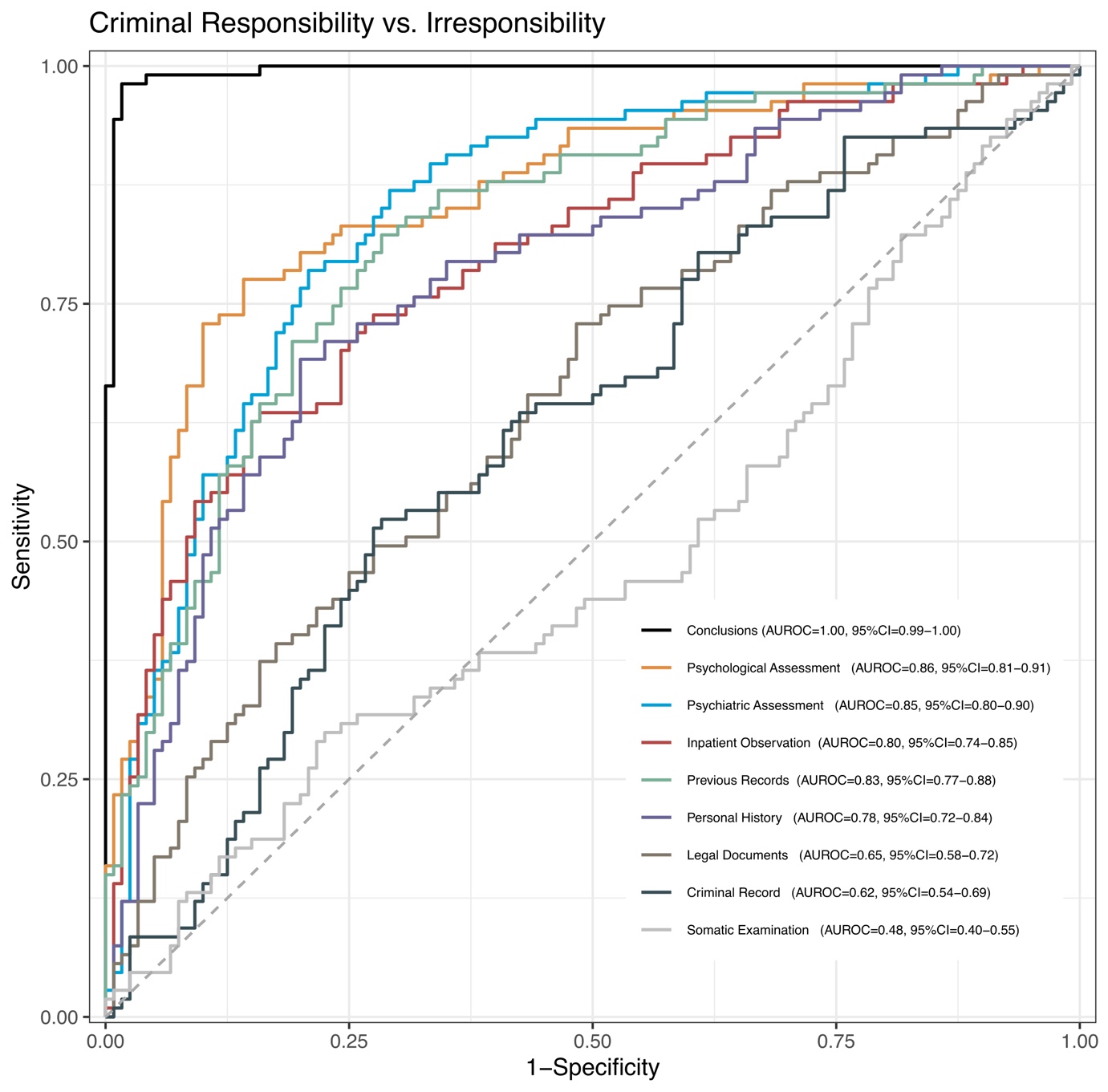
**

**Supplementary Figure 4. Receiver operating characteristic (ROC) curves for Section-Specific Classifiers Distinguishing Criminal Responsibility vs Criminal Irresponsibility via XLM-RoBERTa embeddings as features in SVM.**


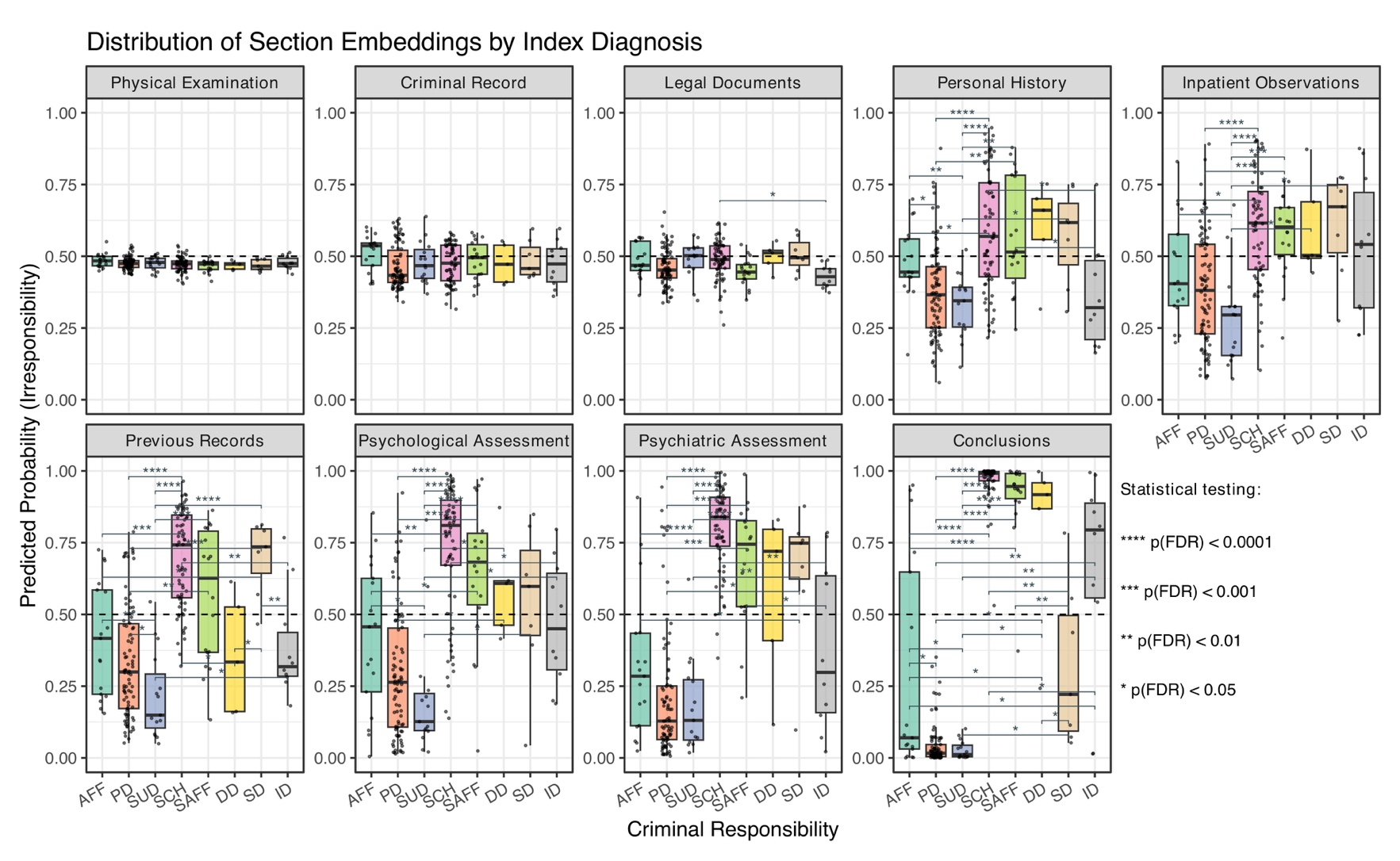


**Supplementary Figure 5.** **Predicted Probabilities of Criminal Irresponsibility by Diagnostic Group across Forensic Psychiatric Assessment Sections.** Boxplots displaying the distributions of the predicted probabilities of criminal irresponsibility derived from section-specific SVM classifiers across diagnostic categories. The dashed line represents a 50% probability (i.e., cutoff between criminal responsibility and irresponsibility). Each panel represents one of the nine sections of the forensic psychiatric assessment (FPA). Higher predicted probabilities indicate greater model-assigned likelihoods of criminal irresponsibility. Diagnostic (index diagnosis) groups are ordered along the x-axis according to increasing clinical responsibility. Abbreviations: AFF, affective disorder; PD, personality disorder; SUD, substance use disorder; SCH, schizophrenia; SAFF, schizoaffective disorder; DD, delusional disorder; SD, schizotypal personality; ID, intellectual disability


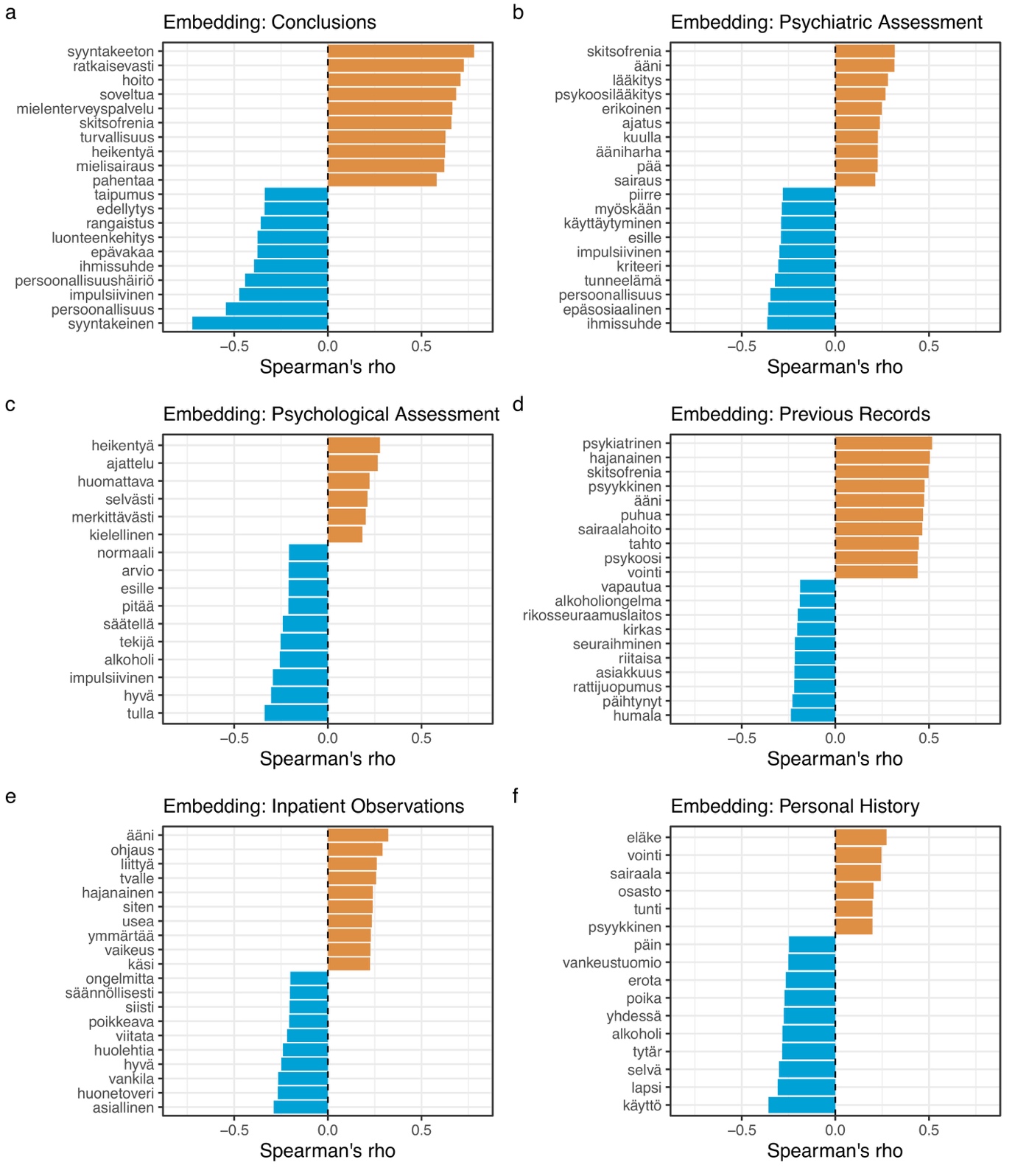


**Supplementary Figure 6. Lemma-Level (original Finnish lemmas) Correlations with Predicted Criminal Irresponsibility across Forensic Psychiatric Assessment Sections.**


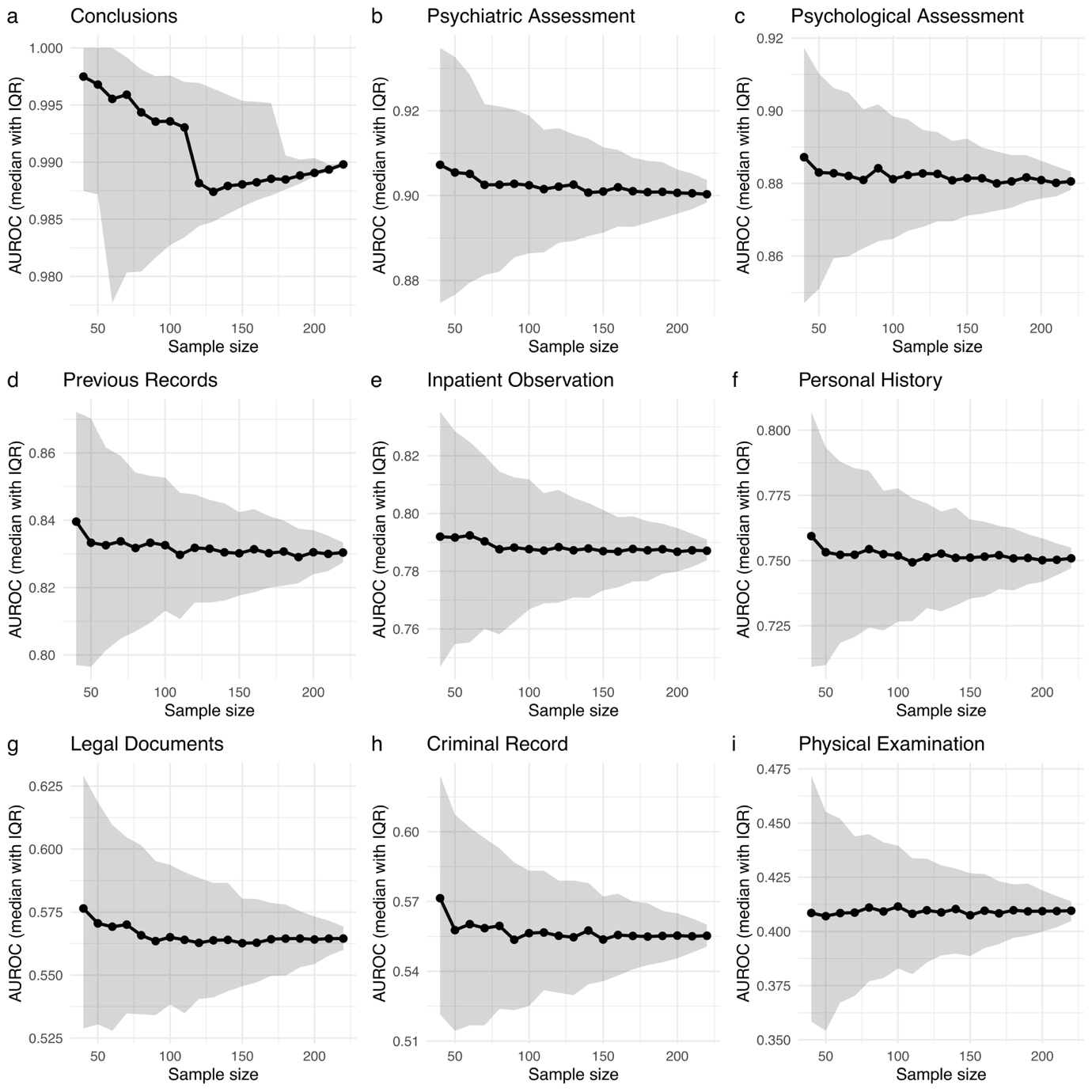


**Supplementary Figure 7. Subsampling-based stability analysis of model discrimination across different sample sizes.**
